# Microarray Analysis of Juvenile Hormone Response in *Drosophila melanogaster* S2 cells

**DOI:** 10.1673/031.010.6601

**Published:** 2010-06-15

**Authors:** David K. Willis, Jun Wang, Joliene R. Lindholm, Anthony Orth, Walter G. Goodman

**Affiliations:** ^1^ USDA/ARS Vegetable Crops Research Unit, University of Wisconsin — Madison, Madison WI 53706; ^2^ Department of Entomology, University of Wisconsin - Madison, Madison WI 53706; ^3^ Genomics Institute of the Novartis Research Foundation, San Diego, CA 92121; ^4^ Current address: INVITROGEN Corporation, 501 Charmany Drive, Madison, WI 53719

**Keywords:** real-time RT-qPCR, *Epac*

## Abstract

A microchip array encompassing probes for 14,010 genes of *Drosophila melanogaster* was used to analyze the effect of juvenile hormone (JH) on genome-wide gene expression. JH is a member of a group of insect hormones involved in regulating larval development and adult reproductive processes. Total RNA was isolated from *Drosophila* S2 cells after 4 hours treatment with 250 ng/ml (10*R*) JH III or 250 ng/ml methyl linoleate. A collection of 32 known or putative genes demonstrated a significant change with JH III treatment (*r* > 2.0, *P* ≤ 0.005). Of these, the abundance of 13 transcripts was significantly increased and 19 decreased. The expression of a subset of these loci was analyzed by real-time quantitative reverse transcription polymerase chain reaction (RT-qPCR). Three loci that exhibited constant expression in the presence and absence of JH III (RP49 [FBgn0002626], FBgn0023529, and FBgn0034354) were evaluated and found to be reliable invariant reference transcripts for real-time RT-qPCR analysis using *BestKeeper* and *geNorm* software. Increased expression in presence of JH III was confirmed by real-time RTqPCR analysis. However, only one of five loci that exhibited reduced expression on microarrays could be confirmed as significantly reduced (P ≤ 0.05). Among the confirmed JH III up-regulated genes were two loci of unknown function (FBgn0040887 and FBgn0037057) and *Epac*, an exchange protein directly activated by cyclic AMP, a guanine nucleotide exchange factor for Rap1 small GTPase.

## Introduction

The insect juvenile hormones (JH) represent a family of acyclic sesquiterpenoids that regulate a diversity of processes in the insect life cycle (Nijhout 1994; Riddiford 1996; Gade et al. 1997; Lafont 2000; Goodman et al. 2005). JH affects insect development by maintaining the larval stage and inhibiting metamorphosis. In adults, JH is involved in regulating reproductive physiology (Riddiford 1996). Although well-studied from the physiological standpoint, the molecular mechanisms underlying JH action remain largely unknown (Jones 1995).

Several molecular mechanisms for JH action have been proposed (Wheeler et al. 2003; Goodman et al. 2005). It has been suggested that JH acts through a specific nuclear receptor complex that modulates gene expression at the level of transcription (Riddiford 1996). This hypothesis is supported by the lipophilic nature of JH and its chemical similarity to the retinoids, compounds known to activate specific nuclear transcription factors, including the vertebrate retinoid X receptor (Mangelsdorf et al. 1995).

Due to its lipophilicity, one might expect the hormone to easily pass through the cellular membrane and interact with cytosolic or nuclear transcription factors; however, there is increasing evidence that suggests JH may act at the membrane level triggering a membranereceptor-mediated signal transduction pathway. In the male accessory glands of *Drosophila melanogaster*, it has been demonstrated that JH acts via protein kinase C and calcium to stimulate protein synthesis (Yamamoto et al. 1988). This interaction with protein kinase C is a classical signal transduction pathway that involves membrane receptors and G-coupled proteins (Sevala et al. 1989; Pszczolkowski et al. 2005; Kethidi et al. 2006). Thus, JH may regulate gene expression at multiple levels and through multiple mechanisms.

Genome-wide gene expression analysis by microarray is the method of choice to identify insect genes that are affected by JH treatment. *A Drosophila* microarray chip is currently available that contains probes for 14,010 putative open reading frames (ORF) within the genomic DNA of this model insect (Affymetrix, Inc). While microarray technology is widely used for expression analysis, the technique exhibits problems that are becoming increasingly apparent. Microarray is a reliable method to detect changes in expression of high abundance genes but the accuracy of identifying changes in low abundance gene transcripts is somewhat problematic (Beckman et al. 2004; Morey et al. 2006). Of particular concern is the ability of microarray analysis to correctly identify changes in low abundance genes or down-regulation of medium abundance genes. Both of these problems arise from the interference of background fluorescence with the low intensity signal from low abundance genes or lower expression of medium abundance genes (Beckman et al. 2004). The accuracy of microarray can be optimized by defining a threshold of reliability based on fold-change and P-value from the chip analysis software, but problems with false positives and false negatives remain (Morey et al. 2006).

Real-time quantitative reverse-transcription quantitative PCR (real-time RT-qPCR) has become the standard technology to verify microarray gene expression profiling. Realtime RT-qPCR has many advantages over microarray for the quantification of specific gene transcripts such as the affordability of performing multiple biological replications and normalizing expression to validated reference RNAs that are known to be invariant under experimental conditions. A major advantage of real-time RT-qPCR is a greatly expanded dynamic range. Microarray analysis can reliably detect expression differences over a three-order of magnitude range (1000-fold) while the dynamic range of real-time RTqPCR extends over seven orders of magnitude (10 million-fold) (Beckman et al. 2004). This enables the accurate measurement of differences over a much larger range of gene expression levels including medium and low abundant transcripts. Two strategies are commonly employed to enumerate the results obtained by real-time RT-qPCR; the standard curve method (absolute quantification) and the comparative threshold method (relative quantification). Absolute quantification relies on the inclusion of a standard curve on each reaction plate and results in determination of the actual quantity of the target transcript expressed in copy number or weight. This method has the advantage of correcting differences in primer efficiencies. The disadvantage of absolute quantification is the significant reduction in the number of experimental samples that can be run on a single plate. Relative quantification determines changes in steady-state mRNA levels of a gene across multiple samples and biological replicates by determining the change in gene expression relative to a control RNA that is designated as the calibrator (Pfaffl 2001; Rasmussen 2001). With this method, target transcript amounts are expressed as a relative expression ratio (RER) relative to the calibrator. Both methods require the normalization of target gene expression using multiple stably expressed internal control mRNAs. These reference gene mRNAs must be shown to be stable under the experimental conditions being examined and are evaluated using software programs such as *BestKeeper* or *geNorm* (Vandesompele et al. 2002; Pfaffl et al. 2004). As with any quantitative measure, care must taken with real-time RT-qPCR to insure that the necessary controls and evaluations have been performed. These include: assessment of RNA quality, assessment of DNA contamination, determination of primer efficiencies and sensitivities, and the use of multiple stable reference RNAs. A recent survey of real-time RT-qPCR publications revealed that only 30% of the published analyses examined satisfied all of these criteria (Bustin 2005).

In this work, we analyzed genome-wide JH III induced expression changes in *Drosophila* S2 cells by microarray. As a control for the lipid component of JH III, methyl linoleate (MLA) was used, as it is a lipid with physical characteristics similar to JH III but is not hormonally active in insects. Microarray expression differences of a select set of genes using real-time RT-qPCR were validated using several reference transcripts that were stably expressed in S2 cells under experimental conditions.

## Materials and Methods

### Purification and quantification of JH homologs

JH III and MLA were purchased from Sigma Chemicals. The biologically active enantiomer (10*R*) of JH III was isolated from a racemic mixture by chiral HPLC chromatography (Cussonetal. 1997).

### Cell culture

*Drosophila* S2 cells (Invitrogen) were maintained in SF900 serum-free medium (Invitrogen) at 27° C. Cells (5×10^5^/ml) were seeded into 60 mm petri dishes (Nunc) containing 3 ml of medium and allowed to grow for 36 h. Cells at approximately 80% confluency were challenged with 250 ng/ml (10*R*) JH III using charcoal-stripped 0.1% bovine serum albumin (BSA) as a carrier for 4 h. Control cells were treated with 0.1% BSA alone or 0.1% BSA with 250 ng/ml MLA and harvested after 4 h of treatment.

### Isolation of RNA

Cells were lysed directly in the culture dishes and total RNA extracted using the RNeasy mini kit (Qiagen). RNA was exhaustively treated with 2 U of Turbo DNAase (Ambion) for 1 h at 37°C and quantified by UV spectrophotometry (NanoDrop, Inc.). RNA quality was determined by electrophoresis of samples on denaturing agarose gels. Residual DNA contamination was quantified using real-time RT-qPCR and primers specific for the *Drosophila rp49* gene (Table 1). Those RNA samples showing threshold cycle (C_q_) values ≥33 cycles were deemed to be free of DNA contamination.

**Table 1.**
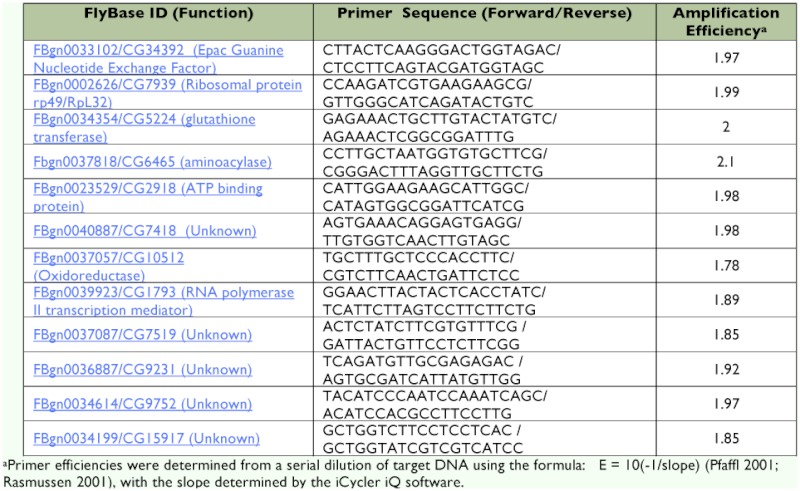
Primers for real-time RT-qPCR

### Microarray analysis

Total RNA was made into cRNA using standard reagents (Affymetrix, Inc). Duplicate total RNA pools from two independently treated S2 cultures were taken for each sample, and resulting single-dye labeled cRNAs were hybridized to *Drosophila* Genome Arrays (Affymetrix, Inc). Arrays were washed with a custom array washer, and scanned with an Affymetrix 3000 scanner. Cell intensity files were analyzed using the Rosetta Resolver algorithm (Rosetta Biosoftware) and comparisons were performed using Resolver's ratio ANOVA function. Resolver ANOVA analysis is similar to standard ANOVA but uses two inputs, expression measurement quantity and estimated error of measurement quantity. This additional input provides more reliable variance measurements, a necessity when the number of replicates is small (Rajagopalan 2003). This error estimate also brings extra degrees of freedom to the analysis, allowing for fewer false positives and false negatives.

### Real-time RT-qPCR primer design

Primers were designed based on *D. melanogaster* mRNA sequences obtained from FlyBase that were imported into Beacon Designer software (Premier Biosoft International); a program designed to generate primer pairs suitable for real-time RT-qPCR. The SYBR Green module with program setting ‘avoid template structure’ was chosen to limit primer sequences to regions of little secondary template structure. Primers were obtained from IDT (Integrated DNA Technologies) and their sequences are shown in Table 1. Both reference and target primers exhibited comparable efficiencies as determined using a dilution series of target DNA. Primer efficiencies were determined from dilution curves using the formula: E = 10^(-1/slope)^ (Pfaffl 2001; Rasmussen 2001), with the slope determined by the iCycler iQ software (Table 1).

### cDNA synthesis and real-time RT-qPCR

First-strand cDNA synthesis was performed using the iScript cDNA synthesis kit according to the manufacturer's instructions (Bio-Rad Laboratories). Briefly, the reaction was performed with 1.0 µg total RNA in 15 µl RNase-free water, 4 µ l 5X iScript reaction mix with a blend of oligo dT and random hexamer primers, and 1 µ l iScript reverse transcriptase. The reaction conditions were performed at 25°C for 5 m, 42°C for 30 m, 85°C for 5 m, and the cDNA was stored at 4°C.

Expression of mRNA was analyzed by realtime RT-qPCR using the iCycler iQ detection system (Bio-Rad Laboratories). Samples were performed in triplicate in 25 µl reactions; 12.5 µl iQ SYBR Green Supermix (Bio-Rad Laboratories), 0.2 µ M forward and reverse primer, and 11.5 µ l of 1:10 diluted cDNA sample. The threshold cycle (C_q_) is the PCR cycle at which the fluorescence of the PCR product exceeds an arbitrary threshold. The C_q_ of the target transcript in RNA from JH IIIchallenged S2 cells was compared with the target transcript C_q_ generated by RNA from S2 cells treated with MLA. Target gene abundance was normalized to three internal reference transcripts that were shown to be invariant using *BestKeeper* (Pfaffl et al. 2004) and *geNorm* (Vandesompele et al. 2002) software. The RER was calculated as the difference between the C_q_ values and was determined using the equation 2^-ΔΔCt^ as previously modified (Rotenberg et al. 2006). PCR conditions were: 95° C for 3 m, 40 cycles of 95° C, 10s; 50° C, 45 s and 1 cycle of 95° C, 1 m; 55° C 1 m then followed by a dissociation curve with 80 cycles of 55° C, 10 s with a 0.5° C increase per cycle. To assure PCR accuracy, PCR reaction products were sequenced directly and compared to the expected target sequence.

Statistical analysis of RER values was performed with GraphPad Prism software using the unpaired two-tailed t-test function (GraphPad Software, Inc).

## Results

### Analysis of JH III effect on genomic expression in *Drosophila* S2 cells.

**Figure 1.**
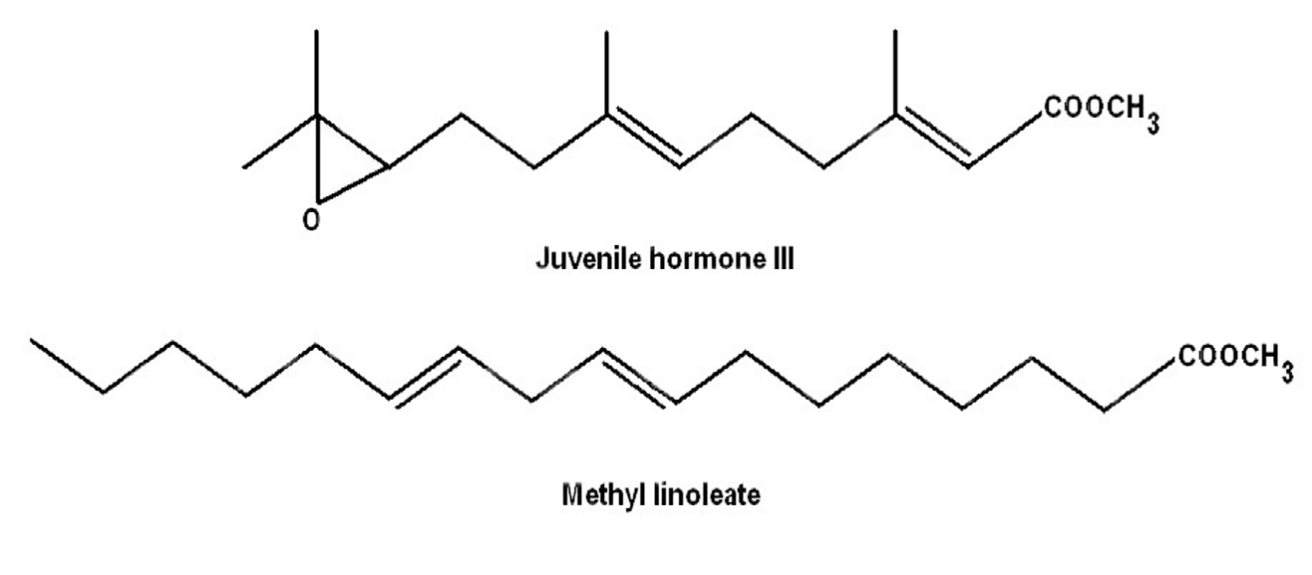
High quality figures are available online.

*Drosophila* microarray chips were challenged with RNA from three treatments of S2 cells: (10*R*) JH III treatment, MLA treatment, or no treatment (no-treatment control). Introduction of lipophilic compounds such as JH III or MLA to culture medium devoid of serum poses a dispersal problem. JH III and MLA are surface active and bind nonspecifically to hydrophobic surfaces (Kramer et al. 1976; Giese et al. 1977). To overcome this problem, we used 0.1% BSA that serves as a carrier molecule to reduce nonspecific binding in all treatments. MLA is a lipid with physical similarity to JH III but lacks hormonal activity. As shown in Figure 1, MLA has a molecular structure comparable to JH III containing two double bonds and an O-methyl ester. In preliminary experiments, MLA was used as a lipid control for JH I due to the related chemical structure and identical molecular weights (WG Goodman, unpublished data). MLA demonstrated no hormonal activity in a *Manduca sexta* bioassay. In addition, JH I but not MLA induced the expression of hemolymph juvenile hormone binding protein mRNA in *M. sexta* when analyzed by real-time RTqPCR. MLA was found to have no effect on *D. melanogaster* eclosion success (JR Lindholm and WG Goodman, unpublished). In the present work, MLA was used to control for any potential effects on gene expression caused by the non-specific cellular metabolism of the JH III added to the S2 cells. Comparing genomic expression from JH III treated cells to control cells (Appendix 1 available online) resulted in numerous putative ORFs showing differences. The following criteria were used to identify potentially significant changes between the two treatments: differences in expression ≥ 2--fold and P-value ≤ 0.01. Using these criteria, 120 of 14,010 (0.86 %) putative *Drosophila* genes demonstrated differences in expression with 14 ORFs showing an increased expression and 106 ORFs a reduced expression (Appendix 2 available online). Comparing MLA-treated S2 cells to the notreatment control (Appendix 3 available online) revealed that 63 of 14,010 (0.45%) ORFs displayed significant changes including 10 up-regulated genes and 53 down-regulated genes (Table 2). Comparing RNA from JH IIItreated S2 cells to MLA-treated cells (Appendix 4 available online) reduced the number of ORFs demonstrating significant differences as only 32 of 14,010 (0.23%) putative *Drosophila* genes exhibited significant differences with 13 genes showing a > 2-fold increase and 19 ORFs showing a decreased expression (Table 3).

Most of the JH III up-regulated genes relative to MLA were loci of unknown function (Table 3). However, *Epac* (FBgn0033102), a gene encoding a guanine nucleotide exchange factor of Rap1 small GTPase, showed a >3fold increase in expression. FBgn0036313 (a serine/threonine kinase) was induced ∼3-fold as were several ORFs with unknown function (FBgn0040887, FBgn0037057, and FBgn0040 603). Among the down-regulated genes were heat *shock protein 70* (FBgn0023529), a transcription factor (FBgn0039923), and *cecropin A2* (FBgn0000277). A large (-5 to 15-fold) down-regulation of the *Drosophila* 18S rRNA was apparently affected by JH III.

**Tabla 2.**
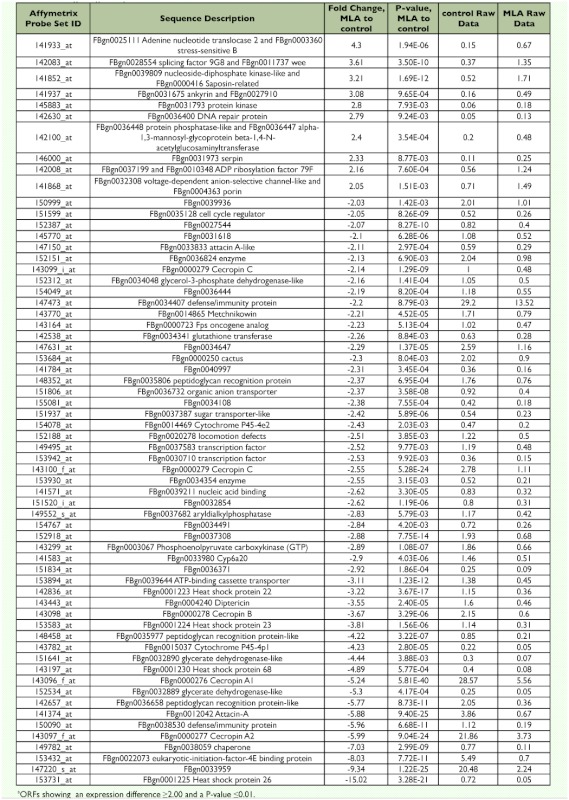
Changes in gene expression between MLA-treated and no-treatment control^a^

**Table 3.**
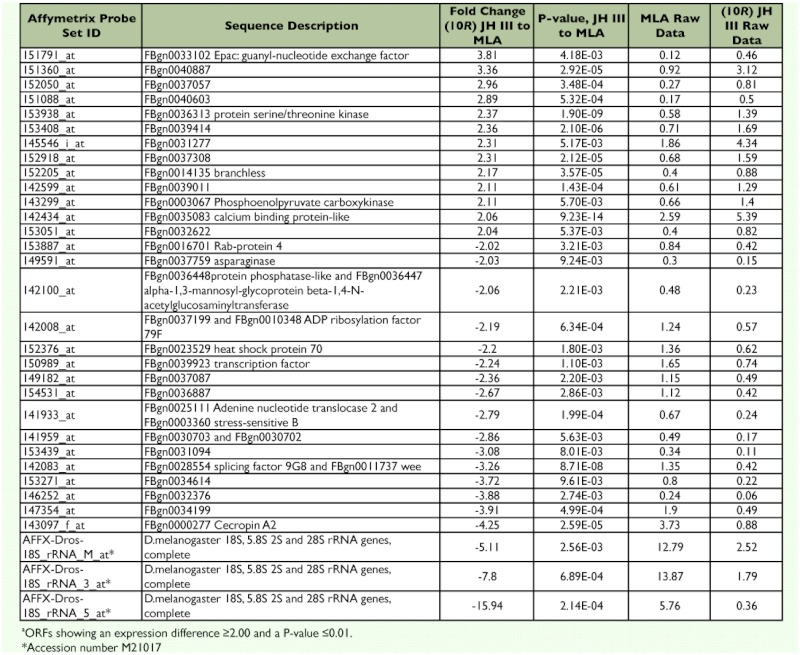
Changes in gene expression between (10R) JH III-treated vs. MLA-treated S2 cells^a^

### Verification of microarray analysis

To verify the expression levels derived from the microarray analyses, *Drosophila* S2 cells were treated with (10*R*) JH III (250 ng/ml) or MLA (250 ng/ml) for 4 h and analyzed for target transcript RER using real-time RTqPCR. Suitable internal reference gene primers were chosen based on genes that were unaffected by the addition of JH III in the microarray analyses (Appendix 4 available online). These were FBgn0023529, which has ATP binding activity and is involved in response to stress, FBgn0034354, which is a glutathione transferase involved in a toxin defense response, and FBgn0002626 (ribosomal protein *49/RpL32*), which is one of the most commonly employed standards used to normalize gene expression in *Drosophila*. Two criteria were used for reference gene characterization: i) primer efficiencies close to 2.00 (100% efficient); and ii) stable expression in total RNA from MLA-treated and JH III-treated S2 cells. Both reference and target primers exhibited comparable efficiencies as determined using a dilution series of target DNA derived from *D. melanogaster* (Table 1). Reference transcript stability was determined using the *BestKeeper* (Pfaffl et al. 2004) and *geNorm* (Vandesompele et al. 2002) programs. *BestKeeper* is an Excel-based tool designed to determine the correlation between the raw values of real-time RT-qPCR for a particular internal reference gene of interest and the geometric mean (the *BestKeeper Index*) of all of the reference genes tested under various treatments. The program performs pairwise Pearson correlations between the C_q_ values of a candidate gene and the *Bestkeeper Index* and reports the measure of the strength of the relationship as an r-value. Ultimately, a strong and significant (*P* < 0.05) correlation (r-value) between the index and the reference RNA candidate determines its stability. The *BestKeeper Index* values were determined from a data set consisting of C_q_ values of potential reference transcripts from both treatments (i.e. multiple RNA samples from MLA-treated and JH III-treated S2 cells). The stability of the reference genes *rp49/RpL32*, FBgn0023529, FBgn0034354 and transcripts (as defined by their respective primer sets) was high (0.96 > r > 0.73). However, the FBgn0034354 transcript consistently exhibited the lowest correlation to the *BestKeeper Index* and therefore was the least consistent of these three reference RNAs. The raw expression data from each internal reference gene was also analyzed using *geNorm* software (Vandesompele et al. 2002). Average expression stability (M) for the reference genes was less than 0.14, which indicates a high degree of constancy under our experimental conditions. Further, *geNorm* analysis of the optimal number of potential internal reference genes suggested that three reference genes were appropriate for data normalization. We have empirically confirmed the stability and utility of these reference transcripts by calculating the RER of several JH III induced genes individually with all three reference genes and found no statistically significant difference in the RERs (data not shown).

The RER was calculated for several of the loci that were significantly changed upon treatment with JH III (Table 3). For this analysis, RNA was isolated from three independent (10*R*) JH III- or MLA- treated cell cultures. Target gene RNA abundances were normalized to the *rp49/RpL32* reference transcript. The RER was calculated using control (MLA treatment) RNAs as a calibrator. The mean of normalized target gene abundance from all three MLA-treated samples was calculated and this value was designated as the ‘calibrator’. Next, the individual target gene abundances from both the JH III- and MLA-treated samples were divided by the calibrator. This allowed the calculation of a RER for each sample replication which was used to compare the means of the treatment RERs statistically. Relative to the MLA-treated cells, the RER of all three JH III up-regulated transcripts were confirmed to be significantly increased (Table 4). *Epac* and two loci of unknown function (FBgn0040887 and FBgn0037057) were tested. In contrast, only one of six genes (FBgn0034199) indicated to be down regulated by the microarray data was confirmed to be significantly reduced by real-time RT-qPCR (Table 4). *Heat shock protein 70* (FBgn0023529) was predicted to be reduced 2.2-fold by JH III action from microarray data but was found to be increased 1.35-fold by real-time RT-qPCR analysis. The remaining four predicted downregulated transcripts were not found to be statistically different (P > 0.05) from the MLA control by two-tailed t-test (Table 4)

**Table 4.**
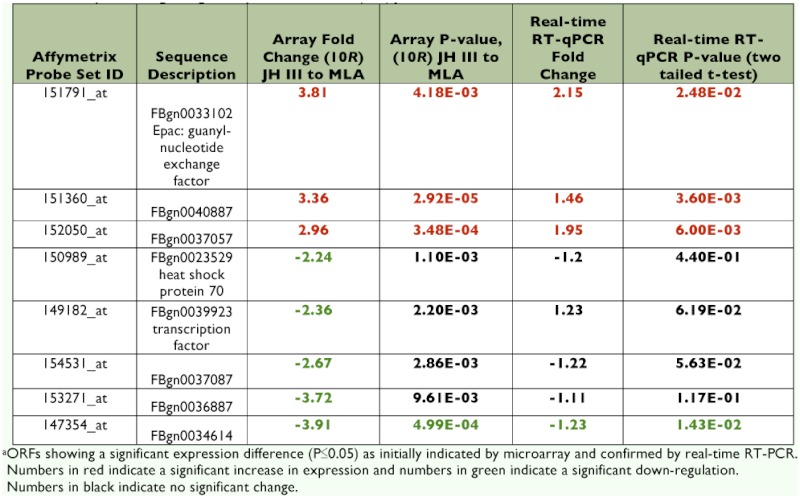
Analyis of changes in gene expression between (10*R*) ]H III treated and MLA-treated control^a^

## Discussion

Microarray analyses indicated that the expression of a number of genes was modified positively or negatively in response to a JH III challenge of *Drosophila* S2 cells (Appendix 1); however, when MLA was tested under identical conditions, expression levels for many genes displayed a similar profile for both JH III and MLA (Appendices 1 and 3).

Less than a 0.5% of the genes examined by microarray analyses demonstrated a highly significant altered level of expression in the presence of JH III but not in the presence of MLA (Appendix 3, Table 4). The up-regulated genes included *Epac*, several loci of unknown function, a serine/threonine kinase, *branchless*, and *phosphoenolpyruvate carboxykinase*. Down-regulated genes included several genes of unknown function, a pre-mRNA splicing factor, *cecropin A2* and *18S rRNA*.

Despite the advantages of microarray technology, real-time RT-qPCR remains the most accurate method to analyze mRNA expression and to verify key relationships identified by microarray analysis (Hembruff et al. 2005). For real-time RT-qPCR to quantitatively assess expression levels of target mRNAs, the selection of appropriate internal reference genes is critical (Vandesompele et al. 2002; Pfaffl et al. 2004; Bustin et al. 2009). It is becoming increasingly evident that reliance on a single reference transcript may lead to significant errors in the analysis of target gene expression (Vandesompele et al. 2002; Pfaffl et al. 2004). Three internal reference RNAs (rp49/RpL32 [FBgn003461 4], FBgn0034354, FBgn0023529) were selected due to their stable expression in S2 cells treated with either JH III or MLA in the microarray analysis. Using three reference genes, as little as a 17% difference in the mean transcript relative expression ratios was shown to be statistically significant (Rotenberg et al. 2006). All three up-regulated genes that we analyzed (*Epac*, FBgn0040887 and FBgn0037057) were confirmed to be upregulated by real-time RT-qPCR (Table 4). However, only one of six genes predicted to be down-regulated by the addition of JH III was confirmed to be statistically reduced by real-time RT-qPCR. This result illustrates the absolute need to confirm microarray data by real-time RT-qPCR analysis before time and resources are expended on further analysis (Morey et al. 2006). The relative inability of microarrays to identify down-regulated genes has been noted previously (Beckman et al. 2004; Morey et al. 2006). This phenomenon relates to the decreased reliability and increased variability in the detection of spots on the microarray exhibiting reduced fluorescence (Beckman et al. 2004).

An important consideration with any expression analysis is the choice of the conditions that will be used as the control for the microarray or real-time RT-qPCR analysis. In this study, we used MLA, a lipid physically similar to JH III but without known hormonal activity, as our control treatment of S2 cells. Another recent microarray analysis of genes that are JH III induced in both *Drosophila* and honey bee, used dimethyl sulfoxide (DMSO), the carrier for JH III in the experiments, as a control treatment (Li et al. 2007). It has been previously shown that DMSO significantly increases juvenoid activity when used as a solvent in a bioassay on *Dysdercus cingulatus* (Sláma 1974). A search of online data (Appendices 1, 2, and 3) revealed that all of the genes described in Li et al. (2007) as JH III inducible were also induced by MLA in our microarray analysis (Appendix 5 available online). This raises the possibility that the induction of these loci may be influenced by the metabolism of the JH III lipid backbone. Due to the increased expression in the MLA control, this set of genes was not identified as JH III inducible (Appendix 5 available online). Since the raw microarray data and the conditions used for microarray analysis in Li et al. (2007) have not as yet been published, the reason for the failure of the previous analysis to identify the genes that were found to be JH III inducible in S2 cells (Table 3, Table 4) is not evident. It may be that DMSO treatment simulates JH III induction to some extent in S2 cells thereby masking the induction, or that this set of genes was induced in S2 cells but not in honey bee by JH III treatment (induction in both insects was a criterion for analysis in the Li et al. 2007 paper). Preliminary real-time RT-qPCR data confirming the specific induction of *Epac* by JH III in both S2 cells and *Drosophila* third instars (Wang et al. 2009) confirms this aspect of our microarray analysis.

In summary, this work details the analysis by microarray of genome-wide gene expression alterations induced by treatment of *Drosophila* S2 cells with JH III. The comparison of JH III treatment to treatment with MLA, a structurally similar but hormonally inactive lipid, revealed only 32 of 14,010 loci responded differentially by microarray analysis. Up-regulated genes were confirmed by real-time RT-qPCR but most predicted down-regulated genes failed confirmation. This indicates that a remarkably small number of genes were specifically affected by JH III. The most intriguing gene that was confirmed to increase expression following JH III treatment was *Epac* that demonstrated highly significant up-regulation in the presence of JH III (≥3-fold) but was refractory to MLA. *Epac*, an exchange factor directly activated by cAMP, is a direct target for cAMP and a guanine-nucleotide exchange factor for the small GTPase, Rap1 (Bos 2005). This suggests that induction of *Epac* expression may be a major component of the JH III hormone's activity in insect development.

## Abbreviations

JH: juvenile hormone;
*Epac*: exchange protein directly activated by cyclic AMP;
MLA: methyl linoleate;
ORF: open reading frame;
RT-qPCR: quantitative reverse transcription polymerase chain reaction;
RER: relative expression ratio
